# Hybridization rate and hybrid fitness for *Camelina microcarpa* Andrz. ex DC (♀) and *Camelina sativa* (L.) Crantz(Brassicaceae) (♂)

**DOI:** 10.1111/eva.12724

**Published:** 2018-12-01

**Authors:** Sara L. Martin, Beatriz E. Lujan‐Toro, Connie A. Sauder, Tracey James, Sara Ohadi, Linda M. Hall

**Affiliations:** ^1^ Ottawa Research and Development Centre Agriculture and Agri‐food Canada Ottawa Ontario; ^2^ Agricultural Food and Nutritional Science University of Alberta Edmonton Alberta

**Keywords:** biosafety, crop‐wild hybridization, gene flow, hybrid fitness, inter‐species hybridization

## Abstract

Hybridization between crops and their wild relatives has the potential to introduce novel variation into wild populations. Camelina (*Camelina sativa*) is a promising oilseed and cultivars with modified seed characteristics and herbicide resistance are in development, prompting a need to evaluate the potential for novel trait introgression into weedy relatives. Little‐podded false flax (littlepod*; Camelina microcarpa*) is a naturalized weed in Canada and the USA. Here we evaluated the hybridization rate between the three cytotypes of littlepod (♀) and camelina (♂), assessed characteristics of hybrids, and evaluated the fitness of hexaploid littlepod and camelina hybrids in the glasshouse and field. In total we conducted, 1,005 manual crosses with diploid littlepod, 1, 172 crosses with tetraploid littlepod, and 896 crosses with hexaploid littlepod. Hybrids were not produced by the diploids, but were produced by the tetraploids and hexaploids at rates of one hybrid for 2,000 ovules pollinated and 24 hybrids for 25 ovules pollinated, respectively. Hybrids between tetraploid littlepod and camelina showed low pollen fertility and produced a small number of seeds. In the glasshouse, hybrids between hexaploid littlepod and camelina also showed significantly lower pollen fertility and seed production than parental lines, but their seeds showed high viability. A similar pattern was observed in field trials, with hybrids showing earlier flowering, reduced biomass, seed production and seed weight. However, seed produced by the hybrids showed greater viability than that produced by hexaploid littlepod and is potentially the result of a shortened lifecycle. The introgression of lifecycle traits into littlepod populations may facilitate range expansion and contribute to crop gene persistence. Consequently, future work should evaluate the hybridization rate in the field, the fitness of advanced generation backcrosses, and the role of time to maturity in limiting hexaploid littlepod's distribution.

## 1 INTRODUCTION

Hybridization between species, and more specifically between crops and their wild relatives, has the potential to cause contrasting effects on the wild population. The introduction of crop alleles, which have been shaped to suit the agricultural environment by artificial selection, are often expected to have negative fitness consequences in natural populations (Jenczewski, Ronfort, & Chèvre, 2003; Stewart, Halfhill, & Warwick, 2003). However, hybridization has played an important role in plant evolution; often through introducing variation that facilitates adaptation. This could create problems by increasing weediness or invasiveness (Ellstrand, 2003; Ellstrand & Hoffman, 1990; Ellstrand & Schierenbeck, 2000; Kwit, Moon, Warwick, & Stewart, 2011; Snow et al., 2010). This may be particularly true for transgenes that confer herbicide resistance (Simard, Légère, & Warwick, 2006; Snow et al., 2010; Warwick et al., 2003; Warwick, Légère, Simard, & James, 2008), but may also be true for other typical crop characteristics such as the production of heavier seeds or shorter time to maturity. Experimental hybridizations between crops and their wild relatives provide a measurement of their baseline inter‐fertility for risk assessment (FitzJohn, Armstrong, Newstrom‐Lloyd, Wilton, & Cochrane, 2007; Garcia‐Alonso et al., 2006), but also provide insights into how hybridization between species could facilitate adaption.

Camelina (*Camelina sativa* (L.) Crantz (camelina) is an oilseed from the Brassicaceae native to Eurasia that has been cultivated for food and lamp oil since the Neolithic (Hovsepyan & Willcox, 2008). Domestication traits in camelina include reduced seed dormancy, larger seed size, determinate flowering and facultative winter/spring annual life cycle. Camelina is receiving renewed attention as a feed, a cosmetic additive, a biofuel and a renewable source of omega‐3 s (Berti, Gesch, Eynck, Anderson, & Cermak, 2016; Betancor et al., 2017; Dangol, Shrestha, & Duffield, 2017; Jiang et al., 2017; Sainger et al., 2017). Transgenic lines of camelina have been developed with modified oil profiles (Jiang et al., 2017; Lu & Kang, 2008) and lines with additional novel traits, including herbicide resistance, are expected to come under considered for unconfined release. *Camelina* also includes weedy flat seeded false flax (*C. alyssum* (Miller) Thellung), little‐podded false flax (littlepod; *C. microcarpa* Andrz. ex DC) and graceful false flax (*C. rumelica* Velen.), which were introduced to North America in the mid to late 1800s (Francis & Warwick, 2009). Littlepod is an agricultural weed and an occasional weed of roadsides and marginal lands in Southern Alberta, Saskatchewan and Manitoba (Francis & Warwick, 2009; Martin et al., 2017) and in the majority of the USA (United States Department of Agriculture, 2016). Until recently (Canadian Government, 2016), camelina species were listed as secondary noxious weeds in the Weed Seeds Order (Canadian Government, [Link]). Hybrids can be produced between camelina and littlepod (Séguin‐Swartz, Nettleton, Sauder, Warwick, & Gugel, 2013) suggesting introgression between the species may be possible.

The genome of camelina indicates that the species is an allohexaploid with 2n = 6*x* = 40 (Hutcheon et al., 2010; Kagale et al., 2014). Littlepod is currently comprised of three cytotypes: a hexaploid (2n = 6*x* = 40) with a DNA content of 1.50 pg/2C, similar to camelina; a tetraploid with 1.00 pg/2C (2n = 4*x* = 26); and a diploid with 0.54 pg/2C (2n = 2*x* = 12) (Martin et al., 2017). Sexual compatibility between camelina and both tetraploid and hexaploid littlepod has already been tested using a small number of crosses (Séguin‐Swartz et al., 2013), but the hybridization rates have not been robustly assessed nor have the hybrids been characterized. Here we examine in detail the ability of all three cytotypes of littlepod to produce hybrids plants when hand pollinated with camelina pollen. While hybridization rate depends on which species is used as the maternal parent (Armstrong, FitzJohn, Newstrom, Wilton, & Lee, [Link]; Ramsey & Schemske, 1998; Thompson, 1930), it is generally considered more likely that pollen will be transferred from high density crop stands to lower density wild populations (Warwick, Beckie, & Hall, 2009). As a result, we choose to prioritize investigation of the potential for hybridization when the wild species is the maternal parent. The hybrids produced between hexaploid littlepod and camelina were investigated with characterization of their reproductive and morphological characteristics, as well as components of their fitness in the glasshouse and the field.

## 2 MATERIALS AND METHODS

### 2.1 Seed sources

Five accessions of camelina were used: two accessions were collected from feral populations in Saskatchewan; one accession “Calena” was supplied by CDA, Lethbridge, Alberta, and two accessions were provided by seed banks: the North Central Regional Plant Introduction Station (NCRPIS) (accession PI258366) and Hortus botanicus, Academia scientiarum, Salaspils, Lativa, URSS in 1987 (accession 3418) (Table 1). For littlepod, diploid seed from NCRPIS (PI650135) and seed from five tetraploid and three hexaploid populations collected in western Canada were used (Table 1).

**Table 1:** Seed sources for littlepod and camelina used in this experiment

Species	Code	Origin	Latitude (N)	Longitude (W)
*Camelina sativa*	CS‐01	Mortlach, Saskatchewan	52° 42.940	108° 22.341
	CS‐02	Estevan, Saskatchewan	49° 22.755	103° 25.139
	CS‐03	North Central Regional Plant Introduction Station Accession ID:PI258366 Collected from Krasnodar Area, Former Soviet Union		
	CS‐04	Hortus botanicus, Academia scientiarum, Salaspils, Lativa, URSS Accession ID:3,418		
	CS‐05	“Calena” Lethbridge, Alberta, Mercer Seeds		
*Camelina microcarpa* 2x	CM2‐01	Lozere, France Accession ID: PI650135		
*Camelina microcarpa* 4x	CM4‐01	Katepwa Beach, Saskatchewan	50°41.378	103°37.063
	CM4‐02	Gainsborough, Saskatchewan	49°10.622	101°26.55
	CM4‐03	Tilston, Manitoba	49°23.515	101°18.999
	CM4‐04	Souris, Manitoba	49°37.502	100°15.471
	CM4‐05	Cromer, Manitoba	49°43.938	101°14.162
*Camelina microcarpa 6x*	CM6‐01	Bow Island, Alberta	49°54.440	111°28.442
	CM6‐02	Lethbridge, Alberta	49°42.382	112°51.732
	CM6‐03	Maple Creek, Saskatchewan	49°54.697	109°28.359

### 2.2 Crossing design

Littlepod seed were stratified in Petri dishes with filter paper moistened with 0.2% potassium nitrate (KNO_3_) wrapped in Parafilm (Pechiney Plastic Packaging Company, Illinois, USA) and placed at 4°C in the dark for 2 weeks. For germination, dishes were placed under growth lights with a 16 hr daylight cycle at room temperature. After cotyledon emergence, seedlings were transplanted into 48 cell trays and placed in growth chambers with a 16 hr photoperiod and 20°C days/18°C nights for 6 weeks. The plants were then vernalized at 4°C with an 8 hr photoperiod, for another 6 weeks. Plants were then transplanted into 20 cm pots and returned to the growth chambers with a 16 hr photoperiod and 24°C days/20°C nights. Camelina pollen donors were raised under the same conditions, but do not require stratification or vernalization. They were planted directly into the 20 cm pots 4 weeks before littlepod was removed from vernalization to synchronize flowering.

Each silicle (pod) of littlepod can contain between 8–25 seeds (Francis & Warwick, 2009). If each flower has at least 10 ovules that can be fertilized, 1,000 crosses would result in 10,000 trials for either success (seed set) or failure (no seed). Using the Poisson distribution, this has the power to detect a hybridization rate of three in 10,000 (0.003%) with 95% confidence and five in 10,000 (0.005%) with 99% confidence (Jhala, Bhatt, Topinka, & Hall, 2011).

The crossing design included four treatments, three controls and crosses between species. Two positive controls were included to indicate our effectiveness as pollinators (a) emasculation and self‐pollination the following day and (b) unmanipulated silicles (pods). Flowers that had only been emasculated served as the negative control and was used to exclude the induction of seed set resulting from manipulation of the flowers. For the hybridization treatment, each of the three accessions of littlepod acted as pollen recipients from each of the five accessions of camelina. Buds were opened, emasculated and then allowed to mature for one day before receiving camelina pollen. As termination of the inflorescence or abortion of pod development and encouragement of thrips movement results from bagging inflorescences, treated plants were left uncovered, well‐spaced, and new buds were removed for three days after treatment. Self‐pollinated and unmanipulated pods from littlepod and were harvested individually and the number of seeds produced per silicle determined. Pods from a single inflorescence that received the emasculation only treatment or the crossing treatment were collected together and total seed production was determined.

### 2.3 Hybrid identification

Flow cytometry was used to screen putative hybrids between camelina and diploid or tetraploid littlepod. As recommended for accurate DNA content, nuclear DNA contents of the parental accessions were determined using replicate samples run across three days with an internal standard (Doležel, Greilhuber, & Suda, 2007). To avoid overlap of the sample and standard peaks, the DNA contents of diploid, tetraploid, and hexaploid littlepod and camelina required that different internal standards were used. Hexaploid littlepod and camelina were co‐chopped with tissue from *Raphanus sativus* L. “Saxa” with a DNA content of 1.1 pg (Doležel, Sgorbati, & Lucretti, 1992). Diploid and tetraploid littlepod were co‐chopped with tissue from camelina which has a DNA content of 1.59 ± 0.05 pg/2C (*n* = 48) (Martin et al., 2017). Fresh tissue from rosettes was collected and kept sandwiched between moist paper towels on ice until chopped with a razor blade in 0.7 ml of Galbraith buffer (Doležel & Bartoš, 2005). Samples were then allowed to stain with propidium iodide for 30–40 min in the dark at 4°C before being run at low speed on a Gallios flow cytometer (Beckman Coulter, Ontario, Canada). Relative DNA content was determined using fluorescence area (585/42 nm detector) and fluorescence peak means, coefficients of variation, and nuclei numbers were measured using ModFit LT software for windows (4.0.5, 2013, Verity Software House Inc., Topsham, ME, USA). The DNA content of diploid and tetraploid littlepod are 0.54 ± 0.02 pg/2C and 1.00 ± 0.02 pg/2C respectively (Martin et al., 2017). Hybrids formed from reduced gametes from each of between diploid and tetraploid littlepod and camelina were expected to have a DNA contents of approximately 1.07 pg/2C and 1.30 pg/2C respectively.

The DNA contents of hexaploid littlepod and camelina are nearly the same (1.50 ± 0.02 pg/2C vs. 1.54 ± 0.05 pg/2C), so species specific molecular markers were used to identify hybrids. Genomic DNA was extracted from fresh tissue using a Fast DNA SPIN kit (MP Biomedicals, Solon, OH USA). The internal transcribed spacer (ITS) region was used to design species specific PCR primers for littlepod and camelina. Separate amplification reactions for each sample included a forward selective primer and a non‐selective reverse primer. The selective forward primers were 16F (5’GAACCAACGATCACCACTCC3’) for camelina and 34F (5’TGATCCCGTTGCCTGCCGTC3’) for littlepod. A common reverse primer, P4CamR, was used in the amplification reactions (5’TTTTCCTCCGCTTATTGATATGC3’). Amplifications were performed using 0.2 μM of each primer, 2.0 μl 10X PCR buffer, 0.2 μM dNTP's, 0.63 unit of Hotstart Taq polymerase (Qiagen, Toronto, Ontario, Canada) and 0.8 μl of genomic DNA in a total volume of 25 μl. A Mastercylcer epGradient thermal cycler (Eppendorf, Mississauga, Ontario, Canada) was used for PCR under the following conditions: 15 min denaturing at 94°C; 35 cycles of 94°C for 45 s, annealing at 66.5°C for 45 s and 72°C for 50 s; then a final elongation at 72°C for 5 min. Reactions were visualized using a 1% agarose gel stained with ethidium bromide. Hybrids were expected to have the ITS marker from both parents and each putatively hybrid plant was tested for both. The presence of an approximately 700 bps band in both reactions confirmed hybridity.

### 2.4 Characterization and backcrossing of tetraploid littlepod and camelina hybrids

We evaluated morphological characteristics of 11 hybrids and three individuals from each of their parental lines grown simultaneously. We measured cauline leaf length; width and auriculate lobe length; and stem trichome density directly from the plants. Additionally, three flowers and three pods of all plants were collected and preserved in 70% ethanol, photographed using Leica M205C microscope (Leica Microsystems, Wetzlar, Germany) with a Leica DF450 camera (Leica Microsystems, Wetzlar, Germany) and measured using the Leica Application Suite (Leica Microsystems, Wetzlar, Germany). In total, 19 traits were measured for each plant: leaf length, width, and lobe length; number of simple and forked stem trichomes in a 1 cm^2^ area; trichome length; total flower length; petal width; sepal length; longest anther length; full gynoecium length; stigma length; and silicle morphology (beak length; pod length, width, and four angles chosen to record pod shape; Supporting information Figure S1).

Pollen viability was assessed using acetocarmine staining. Three flowers were collected per plant at anthesis and the pollen of one anther from each flower was stained with 1% acetocarmine and counted on a Leica DM1000 microscope (Leica Microsystems, Wetzlar, Germany) using the 10x magnification objective. Starting at a random position, 200 pollen grains were scored for viability. Pollen was considered viable if it was round to elliptical and stained.

Eight hybrids between tetraploid littlepod and camelina were randomly chosen and pollinated with tetraploid littlepod and camelina pollen following the protocol used to create hybrids. Emasculated and self‐pollinated, emasculation only, and unmanipulated silicles were also collected, except in one case where the plant was largely male sterile and the self‐pollination control could not be completed.

Once a plant had matured, pods that received backcross or control treatments were collected individually, cleaned and counted by hand. The remainder of the plant was placed in a large paper bag until the seed was cleaned using brass soil sieves before counting.

### 2.5 Fitness and characterization of hexaploid littlepod and camelina hybrids–Glasshouse

Putative hybrid seeds between hexaploid littlepod and camelina were germinated as described above for littlepod except that all material was grown in the glasshouse rather than in growth chambers. At the rosette stage DNA was extracted and hybridity was confirmed using the ITS markers and non‐hybrids were discarded. Following vernalization, putative hybrids and littlepod parental plants were removed from the cold cabinet, transplanted into 20 cm pots and placed back into glasshouse conditions. These plants joined camelina that had been directly sown into pots. Plants were placed randomly in a grid pattern on a bench for the fitness trial and re‐randomized every three weeks. An automatic drip system was connected to ensure consistent watering.

To evaluate morphological characteristics, reproductive ability, and fitness of the hybrids, 118 plants were grown concurrently in the glasshouse including six individuals of each littlepod parental accession (*n* = 18), five individuals of each camelina parental accession (*n* = 25) and five hybrids from each parental cross (*n* = 75). Morphological characteristics and pollen viability were measured as described above for the tetraploid littlepod X sativa hybrids. Once all seed pods had matured, the plants were harvested and placed in paper bags. Seed were cleaned first with brass soil sieves and then by hand to remove debris. Total seed production was assessed by determining the weight of three replicates of 100 seeds and then determining the total seed weight. The approximate number of seeds was then calculated from this information except for individuals where fewer than 300 seeds were produced. In these cases, the exact seed number was determined. Twenty seeds per individual were then placed in Petri dishes as described above except that the dishes were left at room temperature for three days and germination without stratification was scored. Dishes with seeds that had not yet germinated were then stratified and scored for germination a week after removal from 4°C.

### 2.6 Fitness and characterization of hexaploid littlepod and camelina hybrids–Field

As with material used in the glasshouse trial, seed of hexaploid littlepod and hybrids were stratified, vernalized and then the seedlings were transplanted into 48 cell trays. Seeds of camelina, were directly sown into 48 cell trays. All plants were hardened, exposed gradually to outdoor conditions, before transplant to increase transplant success.

Experimental plots were established on May 2014 at the Edmonton Research Station (ERS, 53°29′19″N, 113°34′8″W) into rotor‐tilled Eluviated Black Chernozemic soil. Composite samples from the 0–15 cm depth were analyzed to determine pH, organic matter, available N (nitrate), P, K, and S (sulfate). The soil consisted of 17.2% sand, 35.3% silt, 47.3% clay with the pH of 6 and EC of 0.31 OM 10.4. The soil N, P, K, S content was 15, 20, 280 and 5, respectively.

The experiment was designed as a randomized complete block with six replicates. Seedlings of parental species or hybrids (Table 2) were placed randomly within each replicate (block) and transplanted into a specific plot (30 x 30 cm or 0.1 m^2^ area) wherein nine plants were grown within each plot (i.e. all nine plants in a plot are siblings = line). In contrast to the glasshouse trail, only hybrids from crosses between three accessions of camelina and three accessions of littlepod were included resulting in nine cross combinations (Table 2). Phosphorus fertilizer (P_2_O_5_) was added to the soil below each transplant to promote root establishment. The day after transplantation, seedlings were fertilized by hand using a water‐soluble fertilizer (24‐08‐16). Plants were also watered until they established. Plots were hand weeded required during the season to reduce the effect of weed competition. Moreover, flax (*Linum usitatissimum* L., variety Norlin) was seeded around each plot to provide uniform competition.

**Table 2:** Crosses, seed production, hybrid production and coding for hexaploid littlepod and camelina

	Controls			Emasc. and Cross				
	Emasc. Only	Emasc.and Selfed	Unman.	CS−01	CS−02	CS−03	CS−04	CS−05
Crosses collected								
CM6‐01	28	28	15	75	106	65	78	106
CM6‐02	23	33	16	65	65	68	70	65
CM6‐03	11	21	4	31	32	27	15	28
Seed production								
CM6‐01	28	451	253	1,210	1,401	1,034	1,201	1,428
CM6‐02	3	562	343	1,227	1,258	1,321	1,199	1,106
CM6‐03	0	464	88	619	682	594	359	592
Seeds per pod								
CM6‐01	1.0	16.1	16.9	16.1	13.2	15.9	15.4	13.5
CM6‐02	0.1	17.0	21.4	18.9	19.4	19.4	17.1	17.0
CM6‐03	0.0	22.1	22.0	20.0	21.3	22.0	23.9	21.1
Putative hybrids screened								
CM6‐01				93	95	17	73	78
CM6‐02				98	58	96	92	80
CM6‐03				83	81	77	78	97
Confirmed hybrids								
CM6‐01				93	92	17	71	78
CM6‐02				98	56	94	89	80
CM6‐03				83	80	77	73	97
Coding								
CM6‐01				**H**‐**01A**	**H**‐**01B**	H‐01C	H‐01D	**H**‐**01E**
CM6‐02				**H**‐**02A**	**H**‐**02B**	H‐02C	H‐02D	**H**‐**02E**
CM6‐03				**H**‐**03A**	**H**‐**03B**	H‐03C	H‐03D	**H**‐**03E**

Note

The subset of hybrids in bold were used in the field experiment.

Plant survival was estimated by recording the number of plants at the flowering stage (64 days after removal from vernalization or sowing) and at harvest (163 days after removal from vernalization or sowing). At the flowering stage, one plant from each plot was chosen randomly and the plant's height was measured. Plants were hand harvested prior to shattering. All plants in each plot were removed just above the soil surface and were air dried at 15–30°C for a week. Pods were threshed and seeds were cleaned for each individual. Total plant dry weight, number of seeds and seed weight were obtained for all plants in a plot for all hybrid lines separately. However, total dry weight, number of seeds and seed weight for the parental lines were obtained for parents after pooling all the plants in a plot together. Seed germination was tested as for material from the glasshouse.

### 2.7 Statistical analysis

Statistical analyses were conducted in R 3.0.1 (R Core & Team, 2017). Hybridization rates, pollen viability, and seed viability data were analyzed using Kruskal‐Wallis tests as implemented in the pgimess package (Giraudoux, 2013). Seed production rates by hybridization treatment were analyzed using the gls function from the nlme package (Pinheiro, Bates, DebRoy, & Sarkar, 2015), which allows for different variances among treatments in addition to Kruskal‐Wallis tests. Morphological data were analyzed using linear discriminate analysis (LDA) using the MASS package (Venables & Ripley, 2002). The proportion of plants from each type flowering in the glasshouse at 25 day and the field at 64 days were compared using the function prop.test, while 95% confidence intervals were calculated using the Score method (Newcombe, 1998). Fitness trial data from the glasshouse were analyzed using the aov function with type and line within type as fixed effects, while field trial data for biomass, height at flowering, seed count and thousand seed weight were analyzed using the lmer function with type and line within type as fixed effects and block as a random effect. Harvest time, flowering times, and height data for the glasshouse as well as biomass, seed count and seed weight data from the field were log transformed prior to analysis. Residuals were inspected for normality and heteroscedasticity and in a few cases, such as seed production in the glasshouse indicated a non‐parametric approach might more appropriate and, as a result, Kruskal‐Wallis tests were used in addition to aov and lmer methods. As nested terms cannot be included in Kruskal‐Wallis test, data were broken down by type for analysis by line for field and glasshouse data. Additionally, because of the significance of block for the majority of the field data, field data were standardized by block prior to the Kruskal‐Wallis test. The R packages Hmisc (Harrell Jr, 2018), car (Fox & Weisberg, 2011), pylr (Wickham, 2016), extrafont (Chang, 2014), and plotrix (Lemon, 2006) were used for their graphical and data shaping functions.

## 3 RESULTS

### 3.1 Diploid littlepod and camelina

**Figure 1:**
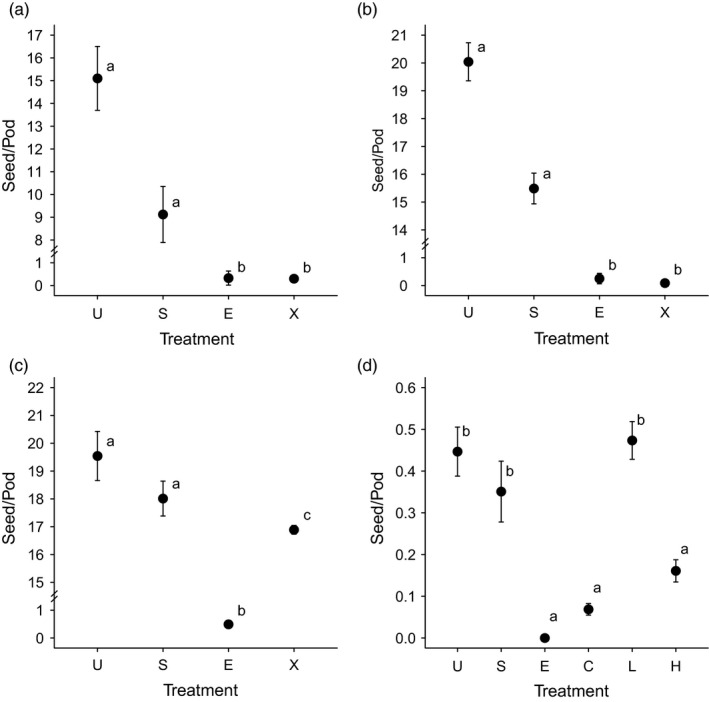
Seed set in (a) diploid littlepod, (b) tetraploid littlepod, (c) hexaploid littlepod, and (d) tetraploid littlepod and camelina hybrids pods that were emasculated only (e), emasculated and self‐pollinated (S), unmanipulated (U) or emasculated and crossed (X) to camelina (a‐c). In addition to these treatments hybrids (d) were back crossed to the parental species (C, L) and crossed to other hybrids (H). Differences among treatments as indicated by a generalized least squares model with allowance for unequal variance among treatments are denoted by lowercase letters. Note axis breaks for plots a‐c

For diploid littlepod and camelina, in addition to 38 unmanipulated pods, we collected 55 emasculation only controls, 57 emasculation and self‐pollination controls, and 1,005 flowers pollinated by camelina. Emasculation only controls produced 0.27 seeds/pod on average, which was significantly lower than the seed production for the emasculation and self‐pollination controls (7.1 seeds/pod) and the unmanipulated pods (17.5 seeds/pod), but not significantly different than the pods that received camelina pollen (0.32 seeds/pod) (Figure 1a; *F*_3,1,128_ = 54.0, *p* < 0.001; χ^2^ = 349, *p* < 0.001). All 296 seeds produced by the crossing treatment were planted. While 107 survived to be assessed using flow cytometry none showed the 2C DNA content expected (data not shown). Given the estimated number of diploid littlepod ovules challenged by camelina pollen was just over 7,000, we had the power to detect hybridization at a rate of 4.2 in 10,000 with 95% confidence. If hybridization occurs between diploid littlepod and camelina, it likely occurs below this rate (Jhala et al., 2011).

### 3.2 Tetraploid littlepod and camelina

For tetraploid littlepod and camelina, we collected 44 emasculation only controls, 84 emasculation and self‐pollination controls, and pollinated 1,172 flowers with camelina pollen following emasculation. In addition, we collected 24 unmanipulated pods. The emasculation only controls produced 0.24 seeds/pod on average, which was significantly lower than the seed production for the emasculation and self‐pollination controls (18.2 seeds/pod) and the unmanipulated pods (20.0 seeds/pod), but not significantly different than the flowers that received camelina pollen (0.08 seeds/pod) (Figure 1b; *F*_3,1,298_ = 540, *p* < 0.001; χ^2^ = 981, *p* < 0.001). In total, 102 seeds were produced by pollinations between the two species. All seeds germinated and survived to screening. Eleven showed an intermediate 2C DNA content averaging 1.23 ± 0.04 2C DNA, while the parental accession of tetraploid littlepod (0.96 ± 0.01 pg/2CDNA; *n* = 5) and camelina (1.64 ± 0.06 pg/2C DNA; *n* = 5) showed 2C DNA contents in line with expectations (Supporting information Table S1). Each cross pollination challenged an estimated 18.1 ovules/flower, indicating a hybridization rate of approximately one hybrid in 2,000 ovules challenged (11 hybrids/21,301 ovules pollinated) between tetraploid littlepod and camelina.

**Figure 2:**
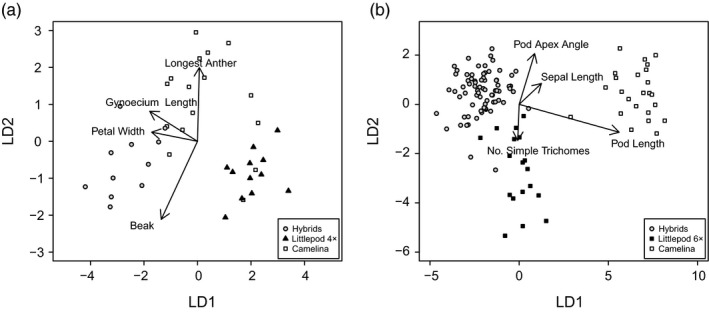
(a) Linear discriminate analysis using six characteristics hybrid (grey circles, *n* = 11), tetraploid littlepod (black triangles; *n* = 15), and camelina (white squares; *n* = 13). The first and second axes account for 80% and 20% of the variance respectively. Contributions of total flower length, petal width and shortest anther length are not shown. (b) Linear discriminate analysis using characteristics hybrid (grey circles, *n* = 75), hexaploid littlepod (black squares; *n* = 18), and camelina (white squares; *n* = 25). The first and second axes account for 89% and 10% of the variance respectively. The contributions of nine additional characteristics are not shown

The hybrids between tetraploid littlepod and camelina more closely resembled camelina than littlepod. Of the 19 characteristics measured, six were retained after the removal of traits with highly correlations (>0.8). Results indicated that the longest anther's length and beak size contributed the most to differentiating among the three types (MANOVA: *F*_12,62_ = 36.6, *p* < 0.001) with the LDA indicating three clusters with some overlap (Figure 2). Acetocarmine staining indicated an average of 18.9% of the hybrid pollen was viable (range 9%–27%; *n* = 11), which was significantly lower (χ^2^ = 30.5, *p* = <0.001) than for either tetraploid littlepod (87.7%, *n* = 15) or camelina (96.5%; *n* = 15). For the eight hybrids chosen for backcrossing to tetraploid littlepod and camelina flowers, the treatments with the highest set seed were as follows: unmanipulated 0.45 seed/pod, self‐pollen 0.35 seed/pod, and littlepod pollen 0.47 seed/pod. Seed set on the emasculation only treatment (0 seed/pod) and camelina pollen (0.07 seed/pod) was significantly lower, while seed production from intra‐hybrid crosses did not differ significantly from either the emasculation only treatment or selfing treatments (0.16 seed/pod; *F*_5,1,262_ = 24.8, *p* < 0.001; χ^2^ = 130.4, *p* = <0.001; Figure 1).

### 3.3 Hexaploid littlepod and camelina

For the crosses between the hexaploid littlepod and camelina, we collected 63 emasculation only controls, completed 82 emasculation and self‐pollination controls, and pollinated 896 buds with camelina pollen. On average the emasculation only controls produced 0.5 seed/pod, which was significantly lower than the seed production for the emasculation and self‐pollination controls (18.0 seed/pod), for the unmanipulated pods (19.5 seed/pod), and the pods that received camelina pollen (16.9 seed/pod). Compared to unmanipulated controls, seed production from the selfing treatment was not statistically different, while the crossing and emasculation only treatments produced significantly fewer seeds (*F*_3,1,072_ = 2,143.4, *p* = <0.001; χ^2^ = 191.6, *p* = <0.001; Figure 1c). Screening all of the seed produced by the hybridization treatment (15,133) was beyond the scope of this study, however, we screened 1,196 putative hybrids. Of these putative hybrids the majority, 98% (1,178), were hybrids with the ITS markers of both littlepod and camelina (data not shown). Assuming this rate of hybridization is consistent, the number of hybrids produced was approximately 14,905. As a result, we estimate the hybridization rate between hexaploid littlepod and camelina is 96% (14,905 hybrids produced by an estimated 15,535 ovules that received camelina pollen; Table 2).

### 3.4 Morphological characteristics of hexaploid littlepod and camelina hybrids in the glasshouse

**Figure 3:**
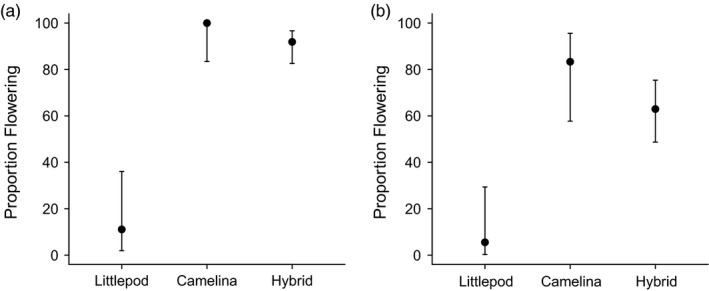
Proportion of plants flowering (a) in the glasshouse 25 days following removal from vernalization for hexaploid littlepod and hybrids and following sowing for camelina and (b) in the field 64 days after removal from vernalization or sowing and their hybrids. Vertical bars indicate 95% confidence intervals

Camelina and hexaploid littlepod have very similar morphology, which leads to difficulty distinguishing between the taxa. Key characteristics that are used to identify the species are the size and shape of the pods, the density and type of trichomes, and seed size. The hybrids were not immediately visually distinct from their parents. However, many individuals had lobed rosette leaves, which contrasted with the entire to wavy leaf margins of the parents. Additionally, the hybrids produced larger, leafier rosettes before vernalization than either parental species. An LDA was conducted on 12 of the morphological traits we measured after the removal of seven traits (pod angle β; leaf length and width; leaf auriculate lobe length; stigma length) that were either very highly correlated (>0.8) with others in the analysis or made small contributions to linear discriminant axes. The first and second axes accounted for 85% and 15% respectively to the total between‐group variance (MANOVA: *F*_30,202_ = 29.48, *p* < 0.0001; Figure 3). In keeping with the characteristics generally used to distinguish between these taxa, the LDA identified pod length, trichome type and the pod's apex angle were the characteristics that accounted for the most variation between groups.

### 3.5 Fitness characteristics of hexaploid littlepod and camelina hybrids in the glasshouse

Within the glasshouse parental species and the hybrids showed significant differences for a number of characteristics (Supporting information Table S2). The proportion of plants flowering at 25 days differed significantly (χ^2^ = 69.24, *p* < 0.001), with hybrid plants flowering an average of 19.5 days (range 15–29 days) following transplant into the glasshouse, which was not statistically different from the 17.5 days (range 12–21) camelina took to flower, but was significantly faster than hexaploid littlepod, which flowered after an average of 31.5 days (range 24–43; Figure 3). However, flowering times of all three types overlapped. The hybrids took longer than camelina to mature with harvest occurring on average 72.8 days following transplant compared to 67 days for camelina (*F*_2,95_ = 107.1, *p* = <0.001). This was faster, but not significantly faster, than littlepod (80.6 days). Lines within type showed significant variation in harvest time (*F*_20,95_ = 4.4, *p* = <0.001) and among hybrids line H‐01A was the last to be harvested (78.2 days), which was significantly later than lines H‐02A (69.2), H‐02D (68.2) and H‐03D (69.4). Plant height at time of flowering did not differ among the hybrids and the parental species (*F*_2,94_ = 2.18, *p* = 0.122), but did differ among lines within type (*F*_19,94_ = 2.66, *p* = 0.001). However, by harvest the hybrids were significantly taller averaging a final height of 144.2 cm compared to 102.7 cm for littlepod and 99.0 cm for camelina (*F*_2,94_ = 262.6, *p* = <0.001). There were also significant differences among lines within types (*F*_20,94_ = 4.5, *p* = <0.001). In all cases, the ANOVAs and Kruskal‐Wallis tests agreed in the significance of type and where line within type was significant in the ANOVAs at least one type had a significant result in the Kruskal–Wallis test (Table S2).

Pollen viability was significantly lower for the hybrids than for camelina or littlepod parental accessions, with an average of 17% (range 1.3%–45%) of compared to 97% and 90% for the parents respectively (χ^2^ = 82.16, *p* = <0.001). While there were no differences among the parental lines of camelina for pollen viability (χ^2^ = 3.31, *p* = 0.51), hybrid lines varied (χ^2^ = 31.37, *p* = 0.005) with both the maternal parent (χ^2^ = 15.19, *p* = <0.001) and the paternal parent showing a significant effect (χ^2^ = 13.22, *p* = 0.01). For example, pollen from hybrids with CM6‐01 as the maternal parent produced more viable pollen (20.7%) than hybrids with CM6‐03 as the maternal parent (12.0%), but they did not differ from those with CM6‐02 as the maternal parent (17.2%).

**Figure 4:**
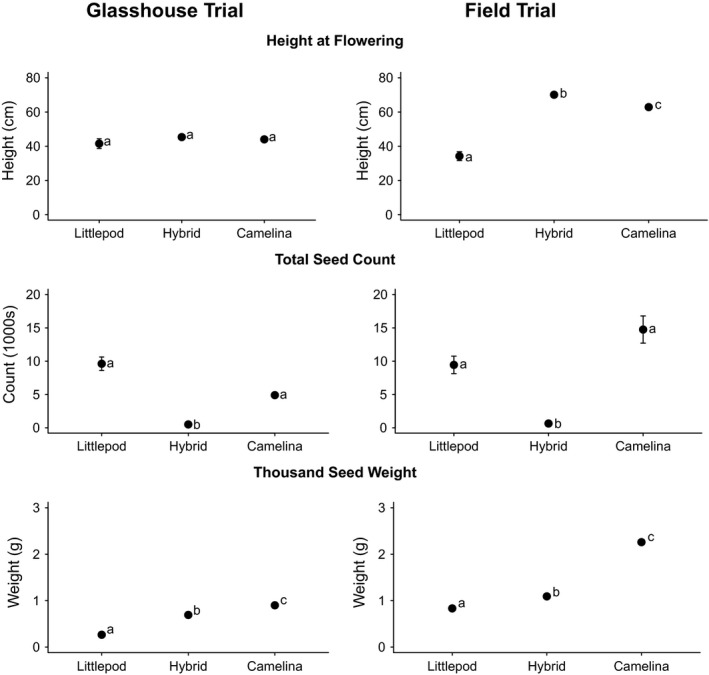
Plant height, number of seeds and thousand seed weight for parental species and hybrids in the glasshouse and field with standard error. Significance indicated based on Kruskal–Wallis tests is indicated in lower case letters

Average seed production was lower for hybrids than for parental accessions at approximately 500 seeds per plant (368.9 mg; range 2–2,618 mg) compared to 9,600 seeds per plant (2,344.4 mg) for littlepod and 4,900 seeds per plant (4,335.6 mg) for camelina (χ^2^ = 12.3, *p* = 0.002; Figure 4). However, there was considerable variation among lines (χ^2^ = 46.32, *p* = <0.001) with both maternal (χ^2^ = 32.54, *p* = <0.001) and paternal parent (χ^2^ = 10.20, *p* = 0.04) significant factors. For example, on average hybrids with CM6‐03 as the maternal parent produced fewer seeds (148) than hybrids with either CM6‐02 (627) or CM6‐01 (742) as the maternal parent.

The seed produced by the hybrids showed high rates of germination (95.9%), which was similar to the rate of germination for the littlepod seeds (99.4%), but less than the complete (100%) germination success camelina seed produced in the glasshouse (χ^2^ = 16.2, *p* = <0.001). All camelina seed, 64.8% of hybrid seed, and 2.2% of littlepod germinated prior to stratification (χ^2^ = 82.5, *p* = <0.001). No camelina seed remained to germinate following stratification, but the remaining 31.1% of the hybrid seed and the majority of littlepod seed (97.2%) germinated after stratification (χ^2^ = 82.4, *p* = <0.001).

### 3.6 Fitness characteristics of hexaploid littlepod and camelina hybrids in the field

When grown in the field, differences among parental species and the hybrids were broadly similar to in the glasshouse, however camelina produced more seed than littlepod in the field. Specifically, while hybrids and littlepod seed production was similar in the glasshouse and field camelina's seed production in the glasshouse was only a third of the production in the field (Supporting information Table S2). One factor contributing to this result is that while the camelina flowering was determinate and seed set was complete by the fall, littlepod flowering was terminated by cool temperatures limiting seed production under field conditions.

Approximately 64 days after the removal of hexaploid littlepod and the hybrids from vernalization and the sowing of camelina seeds into the field, most plants of camelina (83%) and hybrids were in flower (63%), while only one of hexaploid littlepod plants had started flowering (6%; χ^2^ = 25.05, *p* < 0.001; Figure 3). At this stage hybrids were taller (70.1 cm) than camelina (62.9 cm) or hexaploid littlepod (34.2 cm; *F*_2,69_ = 202.1, *p* < 0.001; Supporting information Table S2).

The parental lines of camelina and hexaploid littlepod produced significantly more biomass and more seed than the hybrids, though the weight of the seed produced was intermediate to the parental lines. Camelina and hexaploid littlepod produced more seed on average (camelina: 14,753 seed/plant; littlepod: 9,446 seed/plant) than the hybrids (651 seed/plant; *F*_2_,_484_ = 211.1, *p* < 0.001; Table 3, Figure 4). The viability of this seed differed strongly with the germination rate averaging 86.1% for the hybrids, at 100% for camelina, but only 44.8% for hexaploid littlepod (χ^2^ = 52.7, *p* = <0.001; Supporting information Table S2). Unlike material collected in the glasshouse, the majority of the hexploid littlepod and hybrid seed that germinated, germinated prior to stratification. Specifically, only about 9.9% of the littlepod seed and 6.8% of the hybrid seed germinated following stratification.

**Table 3:** Results of ANOVAs and Kruskal‐Wallis tests for data from the glasshouse with type and line within type and of mixed models with type and line within type as fixed effects and block as a random effect and Kruskal‐Wallis tests following standardization by block for the field data

Location	Trait	Type			Line within type			Kruskal–Wallis by type		Kruskal–Wallis by line within each type					
										Camelina		Hexaploid Littlepod		Hybrid	
		*df*	*F*	*p*	*df*	*F*	*p*	χ^2^	*p*	χ^2^	*p*	χ^2^	*p*	χ^2^	*p*
Glasshouse	Plant Height at Flowering (cm)	2	2.2	0.119	**20**	**2.7**	**<0.001**	1.0	0.56	**10.0**	**0.04**	3.9	0.14	**30.5**	**0.007**
	Plant Height at Harvest (cm)	**2**	**268.4**	**<0.001**	**19**	**5.0**	**<0.001**	**77.8**	**<0.001**	**14.0**	**0.007**	4.6	0.10	**44.4**	**<0.001**
	Number of Seeds	**2**	**209.0**	**<0.001**	**20**	**8.4**	**<0.001**	**12.3**	**0.002**	**13.6**	**0.009**	**12.3**	**0.002**	**46.3**	**<0.001**
	Thousand Seed Weight (g)	**2**	**300.6**	**<0.001**	**20**	**6.6**	**<0.001**	**67.6**	**<0.001**	**16.8**	**0.002**	**11.7**	**0.003**	**46.3**	**<0.001**
Field	Biomass (g)	**2**	**76.8**	**<0.001**	**12**	**13.5**	**<0.001**	**76.3**	**<0.001**	5.4	0.07	**11.3**	**0.004**	**135.5**	**<0.001**
	Plant Height at Flowering (cm)	**2**	**202.1**	**<0.001**	**12**	**3.1**	**0.001**	**50.4**	**<0.001**	**9.2**	**0.01**	**6.3**	**0.04**	**18.1**	**0.02**
	Number of Seeds	**2**	**211.1**	**<0.001**	**12**	**3.0**	**0.004**	**85.1**	**<0.001**	5.3	0.07	5.6	0.06	**20.2**	**0.01**
	Thousand Seed Weight (g)	**2**	**136.3**	**<0.001**	12	1.3	0.20	**77.6**	**<0.001**	1.0	0.62	1.0	0.61	13.8	0.09

Note

Significance at *p* ≤ 0.05 is highlighted in bold face.

Lines within type showed significant differences for all the measured traits except thousand seed weight and in all cases the ANOVAs and the Kruskal‐Wallis tests agreed on the significance of type and line within type. Hybrids varied for biomass, plant height and number of seeds produced. For example, line H‐02A produced on average the most biomass (49.0 g), almost twice the amount of biomass produced by line H‐01B (25.0 g) while line H‐01A produced almost 50% more seed (779) than line H‐03E (529). However, camelina and littlepod lines also differed for these traits (Supporting information Table S2) and, unlike variation by type, line rankings differed between the field and the glasshouse.

## 4 DISCUSSION

Camelina is an emerging oilseed crop with a promising oil profile that is being modified for the food, animal feed and biofuel industries (Berti et al., 2016; Betancor et al., 2017; Dangol et al., 2017; Jiang et al., 2017; Sainger et al., 2017; Small, 2013). The domesticated crop has been introduced to North America as has its congener littlepod (*Camelina microcarpa*). Littlepod is currently comprised of three cytotypes: diploid, tetraploid and hexaploid, with tetraploid and hexaploids populations known to occur within Canada (Francis & Warwick, 2009; Martin et al., 2017). By completing hand crosses between species we evaluated the possibility for transgenes introgression into wild populations prior to unconfined release of these, and assessed the characteristics of crop‐wild hybrids produced, compared to parental lines in the glasshouse and field trials.

As expected, hybridization rate varied with ploidy. We did not detect the formation of hybrids between diploid littlepod and camelina and we estimate our power would allow for the detection of 4.2 hybrids per 10,000 ovules pollinated. In contrast, we detected hybridization between tetraploid littlepod and camelina at a rate of one hybrid per 2,000 ovules pollinated and between hexaploid littlepod and camelina at a rate of 24 hybrids per 25 ovules pollinated, similar to rates reported by Séguin‐Swartz, Nettleton, Sauder, Warwick, and Gugel (2011).

Hybrids between tetraploid littlepod and camelina showed low pollen fertility, in comparison to the parental species, and a limited ability to form seed either through self‐pollination or backcrossing. However, hybrids set more seed when pollinated by tetraploid littlepod than camelina, suggesting that the most likely direction of gene flow between the species would be from the crop species into the wild relative. The hybridization rate between tetraploid littlepod and camelina was low and similar to low rates found for other crosses where the lower ploidy species acted as the maternal parent. For example, here we found a rate hybridization of 0.009 hybrids/flower pollinated, which is similar to the rate (0.004 hybrids/flower) found between shepherd's purse (*Capsella bursa‐pastoris* (L.) Medik) and camelina (Martin et al., 2015). It is also similar to the overall production rate of 0.007 hybrids/pollination for pollinations between canola (*Brassica napus* L.) and 43 other species (FitzJohn et al., 2007). Populations of tetraploid littlepod are apparently less numerous than hexaploid littlepod and occur in less disturbed environments in Canada reducing the area they will overlap with cultivated or feral camelina (Martin et al., 2017) and the potential for introgression.

In contrast, the 16.6 hybrids/pollination produced between hexaploid littlepod and camelina indicate gene flow between the species has a higher probability. Hybrids between hexaploid littlepod and camelina showed reduced fitness in both the glasshouse and the field compared to their parents. For example, in the glasshouse the average pollen fertility and seed production of the hybrids (17% and 500 seeds) was much lower than the parental species (camelina: 97% and 9,600 seed; littlepod: 90% and 4,900 seeds). However, hybrids varied, with ten individuals producing more seed than the least productive littlepod individual. As the hybridization rate estimated here is a worst‐case baseline and the spontaneous rate in the field is expected to be much lower (Walsh et al., 2012). Littlepod species have been described as weedy in the past for example Budd's Flora published in 1979 (Looman & Best, 1979) indicated the species was “fairly common” in fields and waste places, but the weed survey's from the 1970 to 2000’s detected Camelina spp. at low frequency (Leeson et al., 2005). However, we have located large populations of hexaploid littlepod on field margins and waste spaces in the mixed grassland ecoregion of Alberta and Saskatchewan and have described one population that contained both hexaploid littlepod and camelina (Martin et al., 2017). As a result, the species will co‐occur in some regions and hybridization rate should be determined in the field, gene flow among littlepod populations should be assessed, and the fitness of advanced generation hybrids should be investigated.

The fitness consequences of crop alleles introduced into in wild populations is strongly dependent on the genetic, ecological and evolutionary context (Arnold & Hodges, 1995). It has been suggested that genes associated with domestication will generally result in a reduction in fitness for wild relatives (Stewart et al., 2003). However, hybridization and introgression of crop traits in wild populations may result in novel phenotypes that allow for the evolution of weedy biotypes as has been documented in weedy rice, wild radish and wild sunflower (Baute, Kane, Grassa, Lai, & Rieseberg, 2015; Heredia & Ellstrand, 2014; Xia, Wang, Xia, Zhao, & Lu, 2011). Indeed, the alteration of key life history characteristics such as seed dormancy, seedling emergence or flowering time could be beneficial, neutral or deleterious depending on the context. Reduced seed dormancy in hybrids between wild and cultivated sunflowers (Pace, Alexander, Emry, & Mercer, 2015; Snow et al., 1998) and reduced frost tolerance in hybrids between wild and cultivated carrots (Hauser, 2002; Hauser & Shim, 2007) appear to be deleterious. In contrast, earlier emergence may increase invasiveness in hybrid radish as the trait allowed the hybrid to out compete wild radish in a novel environment beyond its range (Hovick, Campbell, Snow, & Whitney, 2012).

Currently, the densest populations of littlepod are found in southern Alberta and southern Saskatchewan where camelina production will be centered with small populations and isolated introductions occurring further north (Martin et al., 2017). The climate in southern Alberta is warmer and the growing season longer than central Alberta where the field trials were conducted (averaging >1,800 growing degree days (GDD) compared to 1,350–1,500 GDD in central Alberta (Alberta Agriculture and Rural Development (n.d.)). North of Calgary, Alberta littlepod appears to occur only as sporadic introductions and is likely beyond the area where self‐sustaining populations can exist (Martin et al., 2017).

Littlepod took almost twice as long to start flowering (31.1 days) than camelina (17.5 days) in the glasshouse and in the field where, 64 days after planting, the majority of the camelina and hybrids were in flower, but only one littlepod had started flowering. In the glass house, littlepod plants were harvested at maturity on average 13.6 days after camelina plants but most seeds were viable (99.4%). In the field, however, littlepod's flowering was terminated by cool temperatures. This appears to have limited seed maturity as less than half of the seeds (44.8%) tested were viable. Littlepod requires a longer growing season for successful seed set and shows the characteristics of an obligate winter annual. The hybrids produced between camelina and littlepod flowered at the same time as the shorter season, annual camelina both in the glasshouse and in the field. The seeds produced by the hybrids in the field showed similar viability to those produced in the greenhouse (95.9% vs. 86.1%) indicating that their seed viability was not strongly limited by the onset of cool temperatures even though the hybrid's time to maturity in the glasshouse (72.8 days) was not significantly reduced compared to littlepod (80.6 days). Further, hybrids show variation for the need for stratification and vernalization indicating that they could introduce this variation into littlepod populations. This variation could allow selection for annual lifecycle and altered seed dormancy in addition to reductions in the length of the growing season required for successful seed set. As a result, hybridization and introgression of these crop traits have the potential to facilitate increased abundance of littlepod in northern portions of its range (e.g. near Edmonton or Peace River) and could allow for the hybrids to outcompete the small littlepod populations we found in these environments. If earlier flowering time is an advantage in wild populations, this could facilitate the persistence of other crop genes including transgenes in these populations and provide an additional example of hybridization facilitating the evolution of a weedy biotype.

## 5 DATA ARCHIVING

The raw data associated with this project are available from the Dryad Digital Repository: https://doi.org/10.5061/dryad.bp6714t.

## CONFLICT OF INTEREST

None declared.
