# Supplementary material for: Hybridization rate and hybrid fitness for Camelina microcarpa Andrz. ex DC (♀) and Camelina sativa (L.) Crantz(Brassicaceae) (♂)
Source: Evol Appl. 2018 Dec 1;12(3):443–55. doi: 10.1111/eva.12724 (PMC6383699; doi:10.1111/eva.12724)
Supplement: Supplementary file 1 [file EVA-12-443-s001.docx]

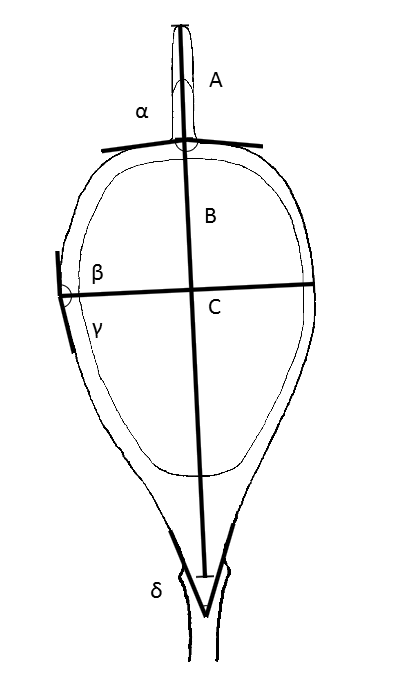
 Figure S1. The four angles, width and length measurements used to characterize pod shape in this study for littlepod, camelina and hybrids. The length and width measurements were A) beak length, B) pod length, and C) pod width at widest point, while the angle measurements were α) angle at pod apex, β) angle at widest point of pod toward beak, γ) angle at widest point of pod toward base, δ) angle at pod base.

Table S2. Characteristics of hexaploid littlepod, camelina and hybrid lines grown in the glasshouse and field with standard deviation.

| Line | | Plant biomass (g) | Plant height at Flowering (cm) | | Number of seeds | | 1000 seed weight (g) | |
| --- | --- | --- | --- | --- | --- | --- | --- | --- |
|  |  | Field | Field | Glasshouse | Field | Glasshouse | Field | Glasshouse |
| Camelina | CS-01 | 96.6 ± 29.7 | 58.0 ± 4.6 | 39.3 ± 1.6 | 9720.8 ± 8185.0 | 5217.0 ± 1260.3 | 2.22 ± 0.002 | 0.93 ± 0.09 |
|  | CS-02 | 142.1 ± 26.6 | 63.0 ± 3.1 | 42.5 ± 2.8 | 19239.8 ± 5743.4 | 5030.2 ± 383.8 | 2.22 ± 0.004 | 1.03 ± 0.04 |
|  | CS-03 |  |  | 45.5 ± 7.7 |  | 4319.2 ± 803.5 |  | 0.92 ± 0.05 |
|  | CS-04 |  |  | 44.9 ± 1.1 |  | 6065.2 ± 930.8 |  | 0.69 ± 0.06 |
|  | CS-05 | 115.8 ± 71.3 | 67.7 ± 5.5 | 48.0 ± 3.3 | 15297.2 ± 9983.5 | 3892.6 ± 388.5 | 2.33 ± 0.251 | 0.93 ± 0.05 |
|  | **Average** | **118.2 ± 48.3** | **62.9 ± 5.9** | **44.0 ± 4.8** | **14752.6 ± 8652.7** | **4904.8 ± 1074.9** | **2.26 ± 0.150** | **0.90 ± 0.13** |
| Hexaploid Littlepod | CM6-01 | 82.1 ± 30.1 | 41.0 ± 15.7 | 48.5 ± 10.7 | 7855.3 ± 8795.2 | 4473.3 ± 2344.3 | 0.73 ± 0.225 | 0.34 ± 0.03 |
|  | CM6-02 | 160.1 ± 33.8 | 33.8 ± 4.9 | 40.6 ± 3.4 | 11808.2 ± 3666.3 | 11341.5 ± 2351.7 | 0.88 ± 0.003 | 0.23 ± 0.02 |
|  | CM6-03 | 123.4 ± 28.3 | 27.8 ± 5.8 | 35.5 ± 16.2 | 8673.3 ± 2118.3 | 13039.8 ± 1426.2 | 0.89 ± 0.036 | 0.22 ± 0.02 |
|  | **Average** | **119.6 ± 43.2** | **34.2 ± 11.0** | **41.5 ± 12.0** | **9445.6 ± 5576.5** | **9618.2 ± 4285.3** | **0.83 ± 0.145** | **0.27 ± 0.06** |
| Hybrid | H-01A | 25.3 ± 14.8 | 61.0 ± 8.1 | 44.4 ± 12.2 | 778.9 ± 647.0 | 843.2 ± 1002.9 | 1.08 ± 0.239 | 0.71 ± 0.09 |
|  | H-01B | 25.0 ± 10.3 | 67.8 ± 3.2 | 45.1 ± 8.5 | 709.9 ± 628.2 | 595.8 ± 600.6 | 1.07 ± 0.219 | 0.65 ± 0.16 |
|  | H-01C |  |  | 51.7 ± 17.2 |  | 911.6 ± 510.1 |  | 0.64 ± 0.07 |
|  | H-01D |  |  | 46.5 ± 9.6 |  | 338.6 ± 310.7 |  | 0.54 ± 0.12 |
|  | H-01E | 25.9 ± 14.5 | 69.3 ± 5.9 | 58.8 ± 15.2 | 607.0 ± 546.9 | 1020.8 ± 1195.5 | 1.11 ± 0.314 | 0.57 ± 0.16 |
|  | H-02A | 49.0 ± 22.8 | 73.0 ± 4.7 | 40.7 ± 4.1 | 769.2 ± 320.9 | 1051.6 ± 183.7 | 1.11 ± 0.207 | 0.79 ± 0.03 |
|  | H-02B | 48.8 ± 20.6 | 72.8 ± 9.8 | 46.6 ± 4.8 | 536.1 ± 269.7 | 665.0 ± 88.6 | 1.04 ± 0.144 | 0.84 ± 0.05 |
|  | H-02C |  |  | 49.2 ± 13.9 |  | 552.6 ± 88.4 |  | 0.77 ± 0.04 |
|  | H-02D |  |  | 50.3 ± 8.6 |  | 353.0 ± 45.8 |  | 0.68 ± 0.09 |
|  | H-02E | 47.3 ± 18.8 | 75.2 ± 3.4 | 45.9 ± 3.8 | 681.8 ± 365.0 | 514.8 ± 66.3 | 1.05 ± 0.165 | 0.75 ± 0.03 |
|  | H-03A | 40.9 ± 21.1 | 71.8 ± 3.9 | 36.8 ± 1.8 | 591.8 ± 335.7 | 276.8 ± 56.4 | 1.17 ± 0.223 | 0.78 ± 0.07 |
|  | H-03B | 45.6 ± 22.2 | 70.7 ± 4.7 | 35.6 ± 1.6 | 567.2 ± 303.6 | 279.6 ± 52.3 | 1.08 ± 0.166 | 0.74 ± 0.13 |
|  | H-03C |  |  | 52.0 ± 12.2 |  | 143.0 ± 49.6 |  | 0.53 ± 0.12 |
|  | H-03D |  |  | 42.2 ± 4.6 |  | 21.8 ± 20.1 |  | 0.71 ± 0.10 |
|  | H-03E | 42.0 ± 14.9 | 69.8 ± 3.4 | 34.7 ± 4.2 | 529.3 ± 235.4 | 21.6 ± 16.3 | 1.09 ± 0.253 | 0.66 ± 0.13 |
|  | **Average** | **38.5 ± 20.5** | **70.1 ± 6.4** | **45.4 ± 10.8** | **645.7 ± 442.4** | **506.0 ± 533.1** | **1.09 ± 0.224** | **0.69 ± 0.13** |
